# The role of risk preferences: voluntary health insurance in rural Tanzania

**DOI:** 10.1186/s13561-023-00432-z

**Published:** 2023-04-01

**Authors:** Alphoncina Kagaigai, Sverre Grepperud

**Affiliations:** 1 Institute of Health and Society, Department of Health Management and Health Economics, University of Oslo, P.O. Box 0315, Oslo, Norway; 2 School of Public Health and Social, Sciences, Department of Development Studies, Muhimbili University of Health and Allied Sciences, P.O. Box 65001, Dar Es Salaam, Tanzania

**Keywords:** Risk preferences, Lottery choices, Health insurance, Medical expenditure risk

## Abstract

**Background:**

Lower-middle-income countries (LMICs) have a common goal to achieve universal health coverage (UHC) through voluntary health insurance schemes. This is important to improve access to healthcare services and ensure financial protection for all by reducing out-of-pocket expenditures. This study aimed to examine the role of risk preferences on enrollment status (currently insured, previously insured, and never insured) into a Tanzanian voluntary health insurance scheme targeted at the informal sector.

**Methods:**

Data were collected from households in a random sample of 722 respondents. The risk preference measure was based on a hypothetical lottery game which applies the BJKS instrument. This instrument measures income risk where the respondents are to choose between a certain income and a lottery. Both multinomial and simple logistic regression models have been used to analyze the relationship between risk aversion and enrollment status.

**Results:**

On average, the respondents have a high degree of risk aversion, and the insured are more risk averse than the uninsured (previously insured and never insured). There is a weak tendency for the wealthiest, measured by household income or total household expenditure, to be somewhat more risk averse than the less wealthy. Logistic and multinomial logistic regressions show that risk aversion is strongly associated with enrollment status. A higher degree of risk aversion significantly increases the probability of being insured, relative to being previously insured, and relative to being never insured.

**Conclusion:**

Risk aversion matters in a decision to enroll into the iCHF scheme. Strengthening the benefit package for the scheme, might increase the enrollment rate and hence improve access to healthcare services for people in rural areas and those employed in the informal sector.

## Background

Risk is an inherent part of decision making, especially so for members of the informal sector in developing economies. Accordingly, it becomes of interest to understand what particular role risk preferences might play. In this paper, we are concerned with assessing the risk preferences for a sample of Tanzanian households and how the distribution of such preferences relates to purchasing power (income and expenditures). Furthermore, we are concerned with the importance risk preferences might have for the decision to enroll or not to enroll into a voluntary health insurance scheme and whether, or not, the inclusion of risk preferences has implications for other explanatory variables (covariates).

The economic literature on risk preferences is typically concerned with the following two research questions: (i) the determinants of risk preferences (attitudes) with a special focus on income and wealth, and (ii) how risk preferences impact decision-making and behavior (choices). In both cases, risk preferences must be estimated where one approach is field studies where environments in which people’s real-world economic behavior is observed [1]. The second is the use of experiments and surveys (questionnaires) to elicit such preferences (non-market behavior). This last approach contains a series of techniques including those that apply lotteries. [2, 3] gives an oversight of various elicitation techniques.

The multiple price list (MPL) techniques, popularized by [4], asks respondents to choose between a sequence of pairwise lotteries (a menu of 10) where each choice is between a safe lottery (where high and low payouts are close) and a risky lottery (the payouts are further apart). For each pairwise lottery, the assigned probabilities are the same across the safe and the risky lottery, however, the probabilities are gradually changed over the lotteries so that the risky lottery becomes increasingly attractive relative to the safe lottery. The number of times the subject chooses the safe lottery in each pairwise lottery is often used as a measure of risk aversion. The MPL technique is widely used, see e.g. [5–9].

Another instrument being frequently used is the one suggested by [10] (the BJKS instrument). This instrument measures income risk and is used in representative samples from several countries. Here the respondents are to choose between a certain income and a lottery. Depending on the response to the initial question, the lottery is changed either upwards or downwards, and the respondents must again choose between a certain income and the revised lottery. Depending on the pair of answers, this instrument classifies the respondents into four different risk categories.

In this work, we apply the BJKS instrument to elicit risk preferences. We are concerned with the distribution of risk preferences and to what extent such preferences differ across enrollment groups and income. The above questions are addressed in connection with a Tanzanian voluntary non-profit insurance scheme—the Community-Based Health Insurance scheme (CBHI). Schemes similar to the CBHI are adopted by several developing countries, often as a response to recommendations given by WHO, but they run under different headings such as Community health insurance [11], Micro health insurance [12], Community health funds [13] and Mutual health organizations [14].

The CBHI scheme of Tanzania was first introduced at the district level in 1996 and the target group was the population living in rural areas and those employed in the informal sector. The scheme was reformed in 2011/2012 by implementing better management systems and by expanding the benefit package [12]. The revised scheme is known as the improved Community Health Fund (iCHF) and was first introduced as a pilot in 6 regions of Tanzania (Dodoma, Shinyanga, Morogoro, Arusha, Manyara and Kilimanjaro). The insurance scheme does primarily provide protection against basic outpatient services meaning that some of the costlier services (inpatient services and medication) are not included unless defined as being qualified for exemptions (pregnant women, elderly and children).

According to [3, 15], there is no consensus on whether risk preferences differ across income and wealth. For western samples, wealthier households (higher annual incomes) are found to display lower levels of risk aversion (examples are Denmark [5], USA [6], Germany [16] and Norway [17]). For developing economies, similar conclusions are arrived at by [18–21], while [7, 22, 23] reach the opposite conclusion. Other studies again, find no relationship between such attitudes and income [24–27].

There is now extensive literature on developing countries that use household survey data to identify associations with insurance enrollment status, however, to the best of our knowledge, this literature does not analyze the role that risk preferences might play. Several systematic reviews on enrollment status confirm this impression. [28] included 25 studies from low-income and middle-income countries published between 2003 and 2013, [29], reviewed 18 studies from sub-Saharan Africa and Asia, published between 2003 and 2013, while [30] reviewed 54 studies, published from 1990 to 2016, mainly from sub-Saharan Africa. The various studies included in the three reviews typically contain socio-demographic variables as independent variables while some, in addition, consider health-related and/or perception variables.

There are, however, studies that consider the role of risk preferences in relation to crop insurance and technology adoption in smallholder agriculture. [31], using survey data from Malawi, finds the adoption of hybrid maize to be lower for farmers who exhibit risk aversion. [32] examined the uptake of crop insurance amongst small scale farmers in India and find that wealthy households are more likely to take up such insurance, while the uptake is lower among credit-constrained households. They also find risk averse households to be less likely to purchase such insurance if they are unfamiliar with insurance in general, or with the microfinance organization offering it. [23], in a study of Chinese farmers, finds that risk averse and loss averse farmers adopt new technologies later in time. [33], studying short-term labor allocation decisions among poor households in Uganda, find that risk preferences and risk perceptions impact household production decisions, particularly for the poorer farmers.

In this study, we extend two previous research papers that apply the same dataset as the one being analyzed here, by adding a variable that measures risk preferences. In the first paper [34], in the following denoted benchmark model 1, a logistic regression model is performed since the dependent variable was dichotomous (insured and uninsured) while the independent variables included socio-demographic variables and perception factors. In the second paper [35], in the following denoted benchmark model 2, multi-nominal logistic regression was performed since the dependent variable had three outcomes (currently insured, previously insured and never insured) while the independent variables now also included health-related variables.

We find, using the BJKS instrument, that our respondents on average are quite risk averse and the insured are more risk averse than the uninsured (never insured and drop-outs), and the previously insured are somewhat more risk averse than the never insured. Second, risk preferences are only weakly correlated with the purchasing power of households in the sense that households with higher incomes and higher total expenditures are somewhat more risk averse. Third, a higher degree of risk aversion, when controlling for a set of variables (socio-demographic, health-related and perceptions), significantly increases; (i) the probability of being insured relative to being uninsured, (ii) the probability of being insured relative to being previously insured, and (iii) the probability for being insured relative to being never insured. Fourth, the inclusion of risk- preferences did not have important effects on the magnitude and direction of other independent variables (covariates).

## Methods

A cross-sectional study design was employed to conduct a household survey in Bahi and Chamwino districts of the Dodoma region in central Tanzania.

### Study setting and sampling

The data for our study were collected through a survey conducted in 2019 for two districts (Bahi and Chamwino) in the Dodoma region of central Tanzania. Administratively, Dodoma is comprised of 7 districts and each district is divided into wards that are subdivided into villages. Bahi is organized into 4 divisions, 22 wards and 59 villages while Chamwino is divided into 5 divisions, 36 wards and 107 villages. The prime economic activity in both districts is agriculture and livestock keeping. According to the National Survey of 2012, Dodoma has a total population of about 2.3 million where 10% live in Bahi and 15% in Chamwino [36].

A multistage sampling technique was used. First, the two districts (Bahi and Chamwino), out of seven, were selected. Second, wards were randomly selected from each district (8 from Bahi and 10 from Chamwino). Thereafter two villages from each ward were selected based on health facility availability and location (16 from Bahi and 20 from Chamwino). We employed systematic random sampling techniques in the selection of households. This was done by starting from the office of the Executive Officer in each village and each interviewer walked in different directions (north, east, south, and west) and selected every third household. The total sample size was 722 households (303 for Bahi and 419 for Chamwino).

All respondents were interviewed face-to-face using a structured pretested questionnaire. The respondents were asked to provide information concerning socio-demographic characteristics, household monthly income and household expenditures. They were also asked about their enrollment status (currently insured, previously insured or never insured) and asked questions relating to the BJKS instrument. The response rate was 100%.

### Variables

#### The risk preference variables

To measure risk preferences each respondent was presented with the questions presented in Table 1. Based on the combinations of answers, each respondent was assigned a value from 1 to 4 (categories) where a higher number refers to a higher degree of risk aversion. Category 4 (Strong) follows if the answer to the conditional sequence of questions (see Table 1) is “job 1” and thereafter “job 1”, for category 3 (Medium) the answers are “job 1” and then “job 2”, for category 2 (Moderate) the answers are “job 2” then “job 1”, while for category 1 (Weak) the answers are “job 2” then “job 2”. This categorical four-scale risk variable is in the following denoted RP4. For subsequent analyses, we also use a dichotomous version of RP4 to measure risk preferences. This variable is constructed by collapsing categories 3 and 4 into one category denoted High and categories 1 and 2 into one category denoted Low. The dichotomous risk preference variable is in the following denoted RP2.

**Table 1:** The BJKS – instrument (the version presented by Schroyen & Aarbu (2018)

Suppose that you are the only income earner in your household. Suppose also that reasons beyond your control force you to change occupation. You can choose between two alternatives. Job 1 guarantees you the same income as your current income. Job 2 gives you a 50% chance of income twice as high as your current income, but with a 50% chance it results in the reduction of your current income by one-third. What is your immediate reaction? Would you choose job 1 or job 2?
If the respondents select the safe alternative (job 1), she is presented with a new pair of alternatives, the only difference being that the downside risk of job 2 is one-fifth of the current income (20% reduction) instead of one-third (33% reduction). If, on the other hand, job 2 is selected, the follow-up question presents the respondent with a choice between the safe alternative and a risky job 2 where the downside risk increases from one-third (33 % reduction) to one-half (50 % reduction).

#### Other independent variables.

Both benchmark models included the following socio-demographic variables; age (4 categories), gender, marital status, household size, and education (3 categories). Both also include household income but they were categorized differently across the two models. In benchmark model 1, income contained 5 categories while in benchmark model 2 income contained 3 categories. In addition, benchmark model 1, in contrast to benchmark model 2, includes religion and occupation as independent variables. As concerning the perception variables, benchmark model 1 consisted of seven variables that were extracted from 38 statements (questions) in the structured questionnaire after subjecting them to principal component analysis (PCA). The questions were formulated as statements and the respondents were asked to express their opinions by using a five-point Likert scale ranging from 1 (strongly disagree) to 5 (strongly agree). In benchmark model 2, however, 5 of the 38 statements were selected as independent variables without undertaking any principal component analysis. The selection of statements was based on previous literature from Tanzania [38, 39] and was concerned with the quality of services, the insurance scheme benefit package, premium affordability, scheme leaders' trustworthiness, and attitudes about traditional healers. Finally, health-related variables were only part of benchmark model 2 and included the following three variables; (i) Chronic diseases (Whether the household had at least one member with a chronic disease or not?), (ii) Fear of sickness (Do you fear the future occurrence of diseases or not?), and, (iii) self-reported health state (EQ-5D) measured by using the EQ-5D instrument which is a generic instrument that uses five dimensions (mobility, self-care, usual activities, pain/discomfort, and anxiety/depression), where each dimension is divided into three levels. The EQ-5D variable was generated as a continuous variable with values ranging from 1 (full health state) to 0 (worst possible health).

### Data analysis

Data were collected using an Open Data Kit (ODK) application and were exported, cleaned, coded, and analyzed using STATA version 17. Data description was done and presented in terms of either frequencies and percentages with a chi square test, or means and standard deviations with a t-test statistic. Results from the logistic regression are presented in terms of odds ratios (OR) (see Table 2). Results from the multinomial logistic regressions are presented as relative risk ratios (RRR) where the currently insured acts as the reference category (base outcome) (see Tables 3 and 4).

**Table 2:** Risk preferences as an enrollment-status determinant: Model 1. Logistic regressions (insured vs uninsured)^a^

Base outcome = Insured		**Benchmark model 1 ** **(Kagaigai et al., 2021)** [34]		**Model 1:RP4**		**Model 1:RP2**	
		OR (95% CI)	P>z	OR (95% CI)	P>z	OR (95% CI)	P>z
**Risk aversion**							
RP4 (Weak = 1)	RP2 (Low =1)						
*Moderate=2*	*High = 2 *	-	-	0.53 (0.19-1.45)	0.22	2.18 (1.38-3.46)	0.00***
*Medium=3*		**-**	**-**	1.22 (0.58-2.53)	0.60	**-**	**-**
*Strong = 4*		**-**	**-**	1.96 (1.17-3.28)	0.01***	**-**	**-**
**Control variables **							
**Socio-demographic variables**							
Age (60+ = 1)							
*40-59*		0.57 (0.33-0.97)	0.04**	0.52 (0.30-0.89)	0.02**	0.53 (0.30-0.91)	0.02**
*26-39*		0.46 (0.26-0.82)	0.01**	0.44 (0.24-0.78)	0.01**	0.44 (0.24-0.79)	0.01**
*18-25*		0.58 (0.24-1.44)	0.24	0.55 (0.22-1.36)	0.20	0.53 (0.21-1.33)	0.18
Income5 (1,000,000+ = 1)							
*0 - 49,990*		0.68 (0.14-3.43)	0.64	0.27 (0.06-1.21)	0.09*	0.26 (0.06-1.13)	0.07*
*50,000 - 99,990*		0.48 (0.12-2.00)	0.31	0.37 (0.08-1.65)	0.19	0.35 (0.08-1.53)	0.16
*100,000 - 499,990*		0.36 (0.08-1.52)	0.16	0.49 (0.11-2.13)	0.34	0.47 (0.11-2.01)	0.31
*500,000 - 999,990*		0.27 (0.06-1.14)	0.08*	0.72 (0.14-3.75)	0.70	0.69 (0.14-3.53)	0.66
Gender (female = 1)							
*Male*		0.75 (0.51-1.10)	0.15	0.75(0.51-1.11)	0.15	0.75 (0.51-1.10)	0.14
Education (Secondary + =1)							
*Primary education*		1.03 (0.55-1.91)	0.93	1.03 (0.55-1.93)	0.93	1.01 (0.54-1.89)	0.97
*No formal education*		1.27 (0.59-2.70)	0.54	1.22 (0.57-2.62)	0.62	1.21 (0.56-2.59)	0.63
Household size (>10 = 1)							
*7-9*		0.76 (0.30-1.93)	0.56	0.81(0.32-2.03)	0.65	0.81 (0.32-2.03)	0.65
*4-6*		0.74 (0.30-1.81)	0.51	0.84 (0.34-2.05)	0.70	0.84 (0.35-2.05)	0.71
*≤3*		0.68 (0.26-1.74)	0.42	0.75 (0.29-1.91)	0.55	0.75 (0.29-1.90)	0.54
Marital status (unmarried = 1)							
*Married*		1.17 (0.76-1.80)	0.49	1.17 (0.75-1.82)	0.49	1.18 (0.76-1.83)	0.46
Occupation (non-farmers = 1)							
*Farmers*		0.95 (0.63-1.44)	0.82	1.20 (0.35-4.10)	0.77	1.25 (0.37-4.25)	0.73
Religion (Muslim = 1)							
*Christian*		1.12 (0.68-1.86)	0.66	1.13 (0.68-1.88)	0.63	1.13 (0.68-1.87)	0.65
**Perception factors**							
*Quality P1*		1.28 (1.10-1.49)	0.00***	1.32 (1.13-1.54)	0.00***	1.31 (1.12-1.53)	0.00***
*Preferences P2*		0.61 (0.52-0.72)	0.00***	0.60 (0.50-0.71)	0.00***	0.60 (0.51-0.71)	0.00***
*Convenience P3*		1.40 (1.17-1.68)	0.00***	1.44 (1.21-1.73)	0.00***	1.44 (1.20-1.72)	0.00***
*Understanding P4*		0.83 (0.72-0.96)	0.01**	0.81 (0.70-0.94)	0.01**	0.82 (0.71-0.94)	0.01**
*Recommendation P5*		0.83 (0.73-0.93)	0.00***	0.81 (0.72-0.92)	0.00***	0.81 (0.72-0.92)	0.00***
*Knowledge P6*		1.39 (1.19-1.62)	0.00***	1.38 (1.19-1.61)	0.00***	1.37 (1.18-1.60)	0.00***
*Awareness P7*		1.08 (0.93-1.24)	0.32	1.06 (0.92-1.23)	0.39	1.06 (0.92-1.22)	0.44

^a^***, ** and * denote significance levels (*p*-value) at 1%, 5% and 10% respectively

**Table 3:** Risk preferences as an enrollment-status determinant: Model 2. Multinomial logistic regressions (never insured vs. currently insured) ^a^

		Benchmark model 2 (Kagaigai et al., 2023) [35]		Model 2.RP4		Model 2.RP2	
Variables		*RRR (95%CI)*	*P* > *z*	*RRR (95%CI)*	*P* > *z*	*RRR (95%CI)*	*P* > *z*
Base outcome (currently insured)							
Risk aversion							
RP4 (Strong = 1)	RP2 (High = 1)						
*Medium* = *2*	*Low* = *2*			0.99 (0.48–2.03)	0.990	3.03 (0,79–11.57)	0.098*
*Moderate* = *3*				3.74 (1.52–9.22)	0.004***		
*Weak* = *4*				2.88 (0.89–9.27)	0.077*		
Control variables							
Socio-demographic variables							
Age (40–59 years = 1)							
18–25		2.33 (1.49–3.65)	0.000***	2.47 (2.04–2.99)	0.000***	2.49 (1.87–3.31)	0.000***
26–39		1.64 (0.50–5.40)	0.418	1.57 (0.51–4.88)	0.433	1.55 (0.49–4.84)	0.448
60 +		0.82 (0.56–1.21)	0.323	0.74 (0.54–1.01)	0.056*	0.75 (0.54–1,04)	0.085*
Income3 (Low = 1)							
*Medium*		0.77 (0.22–2.67)	*0.684*	0.85 (0.31–2.31)	0.753	0.84 (0.32–2.20)	0.727
High		0.57 (0.11–2.85)	0.495	0.72 (0.19–2.63)	0.621	0.72 (0.19–2.59)	0.613
Gender (Male = 1)							
Female		0.52 (0.30–0.89)	0.018**	0.51 (0.30–0.85)	0.009**	0.51 (0.31–0.85)	0.010**
Education (no formal edu = 1)							
Primary education		0.84 (0.84–0.85)	0.000***	0.82 (0.81–0.84)	0.000***	0.82 (0.77–0.88)	0.000***
Secondary educ. +		0.72 (0.68–0.77)	0.000***	0.69 (0.55–0.86)	0.001***	0.68 (0.54–0.86)	0.001***
Household size (1–3 = 1)							
4–6		1.02 (0.67–1.56)	0.926	0.96 (0.63–1.46)	0.862	0.96 (0.67–1.39)	0.846
7–9		0.85 (0.43–1.65)	0.622	0.86 (0.41–1.79)	0.682	0.86 (0.45–1.62)	0.632
10 +		0.74 (0.47–1.16)	0.184	0.87 (0.47–1.62)	0.654	0.71 (0.41–1.85)	0.708
Marital status (unmarried = 1)							
*Married*		0.75 (0.49–1.14)	*0.178*	0.73 (0.47–1.12)	0.156	0.73 (0.47–1.15)	0.173
Health-related variables							
EQ-5D		2.62 (0.23–0.45)	0.441	2.34 (0.17–33.12)	0.529	2.34 (0.18–30.78)	0.517
Fear of sickness (No = 1)							
*Yes*		1.43 (0.41–4.96)	0.572	1.41 (0.44–4.47)	0.560	1.42 (0.45–4.52)	0.551
Chronic diseases (No = 1)							
Yes		0.81 (0.69–0.95)	0.010***	0.82 (0.80–0.85)	0.000***	0.82 (0.77–0.87)	0.000***
Perception variables							
Quality of care		0.68 (0.53–0.87)	0.003***	0.64 (0.47–0.88)	0.007***	0.64 (0.46–0.89)	0.009***
Benefit-premium ratio		0.93 (0.85–1.01)	0.065*	0.93 (0.86–1.01)	0.066*	0.93 (0.87–0.99)	0.039**
Premium affordability		0.89 (0.43–1.86)	0.766	0.89 (0.43–1.85)	0.765	0.89 (0.43–1.85)	0.766
Scheme leader trust		0.47 (0.24–0.88)	0.020**	0.46 (0.27–0.79)	0.005***	0.46 (0.27–0.78)	0.004***
Traditional healers		1.84 (1.19–2.84)	0.006***	1.96 (1.37–2.79)	0.000***	1.95 (1.39–2.73)	0.000***

^a^***, ** and * denote significance level (*p*-value) at 1%, 5% and 10% respectively

**Table 4:** Risk preferences as an enrollment-status determinant: Model 2. Multi-nominal regressions (previously insured vs. currently insured)^a^

		**Benchmark model 2** **(Kagaigai et al., 2023)** [35]		**Model 2:RP4**		**Model 2:RP2**	
Variables		RRR (95%CI)	*P* > z	RRR (95%CI)	*P* > z	RRR (95%CI)	*P* > z
Base outcome (currently insured)							
**Risk aversion**							
RP4 (Strong = 1)	RP2 (High = 1)						
*Medium* = *2*	*Low* = *2*			1.27 (0.86–1.85)	0.223	1.89 (1.38–2.61)	0.000***
*Moderate* = *3*				2.76 (1.20–6.33)	0.017**		
*Weak* = *4*				1.72 (0.99–2.97)	0.053*		
**CONTROL VARIABLES**							
**Socio-demographic variables**							
Age (40–59 years = 1)							
18–25		0.58 (0.21–1.59)	0.289	0.58 (0.23–1.46)	0.246	0.60 (0.22–1.65)	0.321
26–39		1.07 (0.52–2.22)	0.852	1.07 (0.49–2.29)	0.863	1.06 (0.52–2.14)	0.879
60 +		0.66 (0.39–1.10)	0.112	0.62 (0.43–0.89)	0.011**	0.63 (0.41–0.97)	0.036**
Income3 (Low = 1)							
*Medium*		0.64 (0.60–0.69)	0.000***	0.73 (0.69–0.77)	0.000***	0.72 (0.71–0.72)	0.000***
High		0.38 (0.15–0.93)	0.033**	0.51(0.41–0.65)	0.000***	0.51 (0.41–0.63)	0.000***
Gender (Male = 1)							
Female		0.93 (0.48–1.82)	0.838	0.91 (0.45–1.84)	0.791	0.91 (0.46–1.82)	0.797
Education (no formal educatio*n* = 1)							
Primary education		1.10 (1.08–1.12**)**	0.000***	1.06 (1.05–1.08)	0.000***	1.07 (1.05–1.09)	0.000***
Secondary educ. +		0.82 (0.67–1.01)	0.056*	0.81 (0.58–1.13)	0.223	0.79 (0.59–1.08)	0.149
Household size (1–3 = 1)							
4–6		0.97 (0.64–1.46)	0.885	0.95 (0.64–1.39)	0.768	0.95 (0.65–1.39)	0.8
7–9		0.99 (0.36–2.79)	0.998	1.03 (0.36–2.88)	0.968	1.03 (0.38–2.77)	0.96
10 +		0.99 (0.40–2.50)	0.994	1.12 (0.43–2.85)	0.831	1.09 (0.49–2.42)	0.824
Marital status (unmarried = 1)							
*Married*		0.90 (0.51–1.59)	*0.723*	0.91 (0.49–1.67)	0.749	0.89 (0.50–1.60)	0.716
**Health-related variables**							
EQ-5D		1.43 (0.47–4.31)	0.53	1.44 (0.55–3.79)	0.462	1.42 (0.53–3.81)	0.49
Fear of sickness (No = 1)							
*Yes*		1.08 (0.90–1.29)	0.421	1.07 (0.79–1.44)	0.681	1.09 (0.82–1.45)	0.548
Chronic diseases (No = 1)							
*Yes*		0.58 (0.34–0.99)	0.045**	0.58 (0.33–0.97)	0.047**	0.58 (0.33–1.02)	0.057*
**Perception variables**							
Quality of care		0.86 (0.81–0.91)	0.000***	0.83 (0.76–0.91)	0.000***	0.83 (0.75–0.92)	0.000***
Benefit-premium ratio		1.16 (0.85–1.60)	0.345	1.16 (0.86–1.57)	0.328	1.17 (0.85–1.61)	0.342
Premium affordability		0.69 (0.51–0.95)	0.021**	0.69 (0.50–0.96)	0.029**	0.69 (0.51–0.96)	0.026**
Scheme leader trust		0.76 (0.55–1.06)	0.094*	0.76 (0.59–0.97)	0.03**	0.75 (0.57–0.98)	0.040**
Traditional healers		1.20 (1.18–1.23)	0.000***	1.25 (1.24–1.27)	0.000***	1.25 (1.16–1.34)	0.000***

^a^***, ** and * denote significance level (*p*-value) at 1%, 5% and 10% respectively

## Results

### Descriptive statistics

Two Hundred Eighteen of the 722 households were insured (30.1%) while 504 were uninsured (69.9%). Of the uninsured, 395 had previously been insured (dropouts), while the remaining 109 had never been insured by the scheme in question (54.7% and 15.2%, respectively, of the total sample)**.** The average age of the respondents was 44.7 years, there were more females (57.9%), 3 out of 4 were married, the average household size was 5.4 members, the majority were farmers (74%) and 72% had primary education while 18% had no education.

The distribution of observable household characteristics across insured and uninsured are available in Table 5. The two enrollment groups did not differ with respect to education, marital status, household size and occupation, while they differed to some extent for gender and age (females and those belonging to the oldest age groups (+60 years) were more likely to be insured), and differed significantly for the two income variables (Income 5 and Income3) in the sense that those with the highest income were more likely to be insured.

**Table 5:** Socio-demographic characteristics by enrollment status (*n* = 722). Frequencies (%)

Sample characteristics	Insured	Uninsured	Total sample	*P* > Z
*Age (years)*				
18–25	13 (5.9)	29 (5.8)	42 (5.8)	
26–39	63 (28.9)	176 (34.9)	239 (33.1)	0.147
40–59	103 (47.2)	238 (47.2)	341 (47.2)	
60 +	39 (17.9)	61 (12.1)	100 (13.9)	
*Gender*				
Female	134 (61.5)	284 (56.4)	418 (57.9)	0.201
Male	84 (38.5)	220 (43.7)	304 (42.1)	
*Education*				
No education	36 (16.5)	91(18.1)	127 (17.6)	
Primary	154 (70.6)	366 (72.6)	520 (72.0)	0.350
Secondary and higher	28 (12.8)	47 (9.3)	75 (10.4)	
*Marital status*				
Unmarried	55 (25.2)	143 (28.4)	198 (27.4)	0.385
Married	163 (74.8)	361 (71.6)	524 (72.6)	
*Household size*				
≤ 3	40 (18.4)	101 (20.0)	141 (19.5)	
4–6	112 (51.4)	261 (51.8)	373 (51.7)	0.918
7–9	56 (25.7)	122 (24.2)	178 (24.7)	
≥ 10	10 (4.6)	20 (4.0)	30 (4.2)	
*Occupation*				
Non-farmer	53 (24.3)	120 (23.8)	173 (23.9)	0.885
Farmer	165 (75.7)	384 (76.2)	549 (76.0)	
*Income5 (5 categories))*				
0—49,999	66 (30.3)	205 (40.6)	271 (37.5)	
50,000—99,999	59 (27.1)	132 (26.2)	191 (26.5)	
100,000—499,999	76 (34.9)	144 (28.6)	220 (30.5)	0.037 **
500,000—999,999	12 (5.5)	19 (3.8)	31 (4.3)	
1.000,000 +	5 (2.3)	4 (0.8)	9 (1.3)	
*Income3 (3 categories)*				
Low	66 (30.3)	205 (40.7)	271 (37.5)	0.013 ***
Medium	135 (61.9)	276 (54.8)	411 (59.9)	
High	17 (7.8)	23 (4.6)	40 (5.5)	
Total	218 (30.2)	504 (69.8)	722 (100)	

By cross tabulating the socio-demographic characteristics across risk preference groups (RP2), we find that the risk preferences differ significantly with respect to the occupation (*p=*0,013), enrollment status (*p=*0,014) and health state (EQ-5D) (*p=*0,059) and to some extent with respect to mean income and household size (see Table 6). The other variables (age, gender, education and marital status) are not significant.

**Table 6:** Socio-demographic characteristics by risk preferences (RP2) (mean and percentage shares)

Sample characteristics	Low RP	High RP	Total sample	Pr (|T| >|t|) =
	Mean	Mean	Mean	
Income	107,480	128,785	124,359	0.218
*Age (years)*	44.8	44.63	44.67	0.894
*Household size*	5.2	5.44	5.39	0.241
EQ-5D	0.79	0.76	0.76	0.059 *
	**Low RP** **n (%)**	**High RP** **n (%)**	**Total sample** **n**	***P* > z**
*Enrollment status*				
Never insured	30 (27.5)	79 (72.5)	109	0.014 ***
Currently insured	32 (14.7)	186 (85.3)	218	
Previously insured	88 (22.3)	307 (77.7)	395	
*Gender*				
Female	90 (21.5)	328 (78.5)	418	0.557
Male	60 (19.7)	244 (80.3)	304	
*Education*				
No education	27 (21.3)	100 (78.7)	127	
Primary education	105 (20.2)	415 (79.8)	520	0.741
Secondary and higher	18 (24.0)	57 (76.0)	75	
*Marital status*				
Unmarried	41 (20.7)	157 (79.3)	198	0.978
Married	109 (20.8)	415 (79.2)	524	
*Occupation*				
Non-farmer	27 (14.4)	160 (85.6)		0.013 ***
Farmer	123 (22.9)	412 (77.0)		
Total	150 (20.8)	572 (79.2)	722 (100)	

As concerning enrollment status, more than 70% of the respondents in each enrollment group belong to the high risk preference group and the currently insured were significantly more risk averse (85.3%) than the never insured (72,5%) and the previously insured (77.7%). In Appendix A, we also present the distribution of risk preferences across enrollment groups when risk preferences are categorized into 4 groups (RP4). Again the currently insured are on average more risk averse than the previously insured and the never insured, however, now the two uninsured groups do not differ much and the differences are insignificant (*p* = 0.112), possibly being the result of a limited number of observations for one of the risk categories (Moderate).

From Table 6, we also observe that a higher mean household income, to some extent, is associated with higher risk aversion, however, this might be the result of income being correlated with other variables for example occupation and education. In Appendix B, we present the distribution of risk preference, measured by RP4 and RP2, by income3. It follows that more risk aversion, measured by RP4, is associated with higher household income in a significant way (*p* = 0.04). The same pattern matters for RP2, however, now the associations are insignificant (*p* = 0.25).

An alternative to household income as a measure of purchasing power (living standard) is household expenditures [40]. In appendix C, we present the distribution of risk preference across total household expenditure (socioeconomic status). Both for RP4 and RP2, there is a weak tendency for the households in the highest quintile (highest socioeconomic status) to be somewhat more risk averse as compared to households in the lowest quintile (lowest socioeconomic status), however, the overall associations are strongly insignificant (*p* = 0.87 for RP4 and *p* = 0.57 for RP2).

In Table 7 we present the distribution of answers to the BJKS lottery questions together with results from three national surveys (Norway, USA, and Chile) all using the BJKS instrument. Such a comparison enables us to say something about the relative significance of risk-averse preferences for our sample relative to the national samples. The surveys were collected in 2002 for the USA (*n =* 3,591) and Chile (n=11,475) and in 2006 for Norway (*n =* 1,554) (for further details on the three surveys, see [37]). From Table 7 we observe that the distribution is skewed since 2 out of 3 households belong to category 4 (Strong). The same pattern is present for the other three countries although being less pronounced for Norway. Our sample is on average less risk averse than Chileans but more risk averse than Norwegians. When aggregating Weak and Moderate into the category Low and Medium and Strong into the category High, the distributions become as follows; Norway (22.9% vs. 78.1%), the USA (20.6% vs. 79.4%), our sample (20.8% and 79.2%) and Chile (8.8% vs. 91.2%). Hence, the shares for category High are almost the same for Norway, the USA, and our sample (almost 80%), and of these, 81% (the USA) and 84% (our sample) belong to the category Strong while this share for Norway is only 47%. The above discussion suggests that out of the three countries, the distribution of risk preferences for our sample is closest to the one of the USA.

**Table 7:** Comparisons across countries (shares): The BJKS instrument

	Weak	Moderate	Medium	Strong
Our sample (Tanzania) (*n* = 720)	14.1	6.7	10.8	68.4
Norway (*n* = 1,554)	13.3	8.6	41.3	36.8
USA (*n* = 3,591)	11.7	9.6	15.3	63.4
Chile (*n* = 11,475)	4.7	4.1	9.3	81.8

Sample size (*n*). The data sources for the different countries are; Norway (Schroyen & Aarbu, 2018) [37], the USA (Kimball et al., 2008) [41] and Chile (Martinez & Sahm, 2009) [42]

### Regression results

The results from performing the logistic regression analysis are presented in Table 2 while the results from performing the multivariate analysis are presented in Tables 3 and 4. The results presented in the first column of all three tables are models that do not include the stated risk aversion measure as an independent variable (the benchmark models) while the next two columns present the results when adding each of the two risk aversion measures (RP4 and RP2). 

 For benchmark model 1 (see Table 2), we observe that six of the seven perception variables (P1 to P6) and two age groups are significant (5%). For Model 1:RP4 (adding RP4), we observe that the only significant risk preference group is Strong. In this case, the odds of being insured (relative to uninsured), when moving from Weak to Strong, is almost as twice as high (OR=1.96, *p* = 0.01). The odds ratios, when moving from Weak to Moderate and from Weak to Medium, are both insignificant and pull in opposite directions (0.53 vs. 1.22). For the dichotomous risk preference variable (Model 1:RP2), the odds ratio for a higher degree of risk aversion is strong and significant (OR = 2.18, *p* = 0.00). In this case, belonging to High, relative to Low, implies that the odds of being insured (relative to uninsured), are more than twice as high. 

We also observe from the odds ratios and the significance levels of the control variables, in both models, maybe except for income, that they remain stable in response to the introduction of risk preferences. Furthermore, to investigate the role of the control variables, we also conducted bivariate logistic regressions by regressing RP2 on enrollment status. The odds ratio remained significant, but the magnitude became somewhat lower relative to Model 1:RP2 (OR = 1.78 and *p* = 0.008) (see Appendix D).

The next two tables (multi-nominal regression) present the results for the never insured (Table 3) and the previously insured (Table 4), relative to the currently insured. For the benchmark model that concerns the never insured (see Table 3), the significant variables are chronic diseases, one age group (18-25 yrs.), gender, both educational groups, and, four, out of, the five perception variables. As concerning the effects of risk aversion (Model 2:RP4), we find that having a Moderate degree of risk aversion or a Weak degree of risk aversion, compared to a Strong degree of risk aversion, increases significantly the probability of being never insured relative to being currently insured (*RRR= 3.74, p=0.004 and RRR=2.88, p=0.077, *respectively). For the dichotomous risk preference variable (Model 2:RP2), the relative risk ratio (RRR) is significant and of a quite high magnitude (RRR=3.03, *p=*0.098), saying that a low degree of risk aversion, relative to having a high degree of risk aversion, increases the probability of being never insured relative to being currently insured. Both for Model 2:RP4 and Model 2:RP2, the introduction of risk preferences does not change the relative risk ratios and the significance levels of the control variables relative to benchmark model 2, with the exception of one of the age groups (60+ yrs.) that becomes significant at 1% level for both models.

For the benchmark model that concerns the previously insured (see Table 4), the identity of the significant variables differs somewhat from the findings of Table 3. Chronic disease and both educational groups remain significant while age and gender become insignificant. In addition, both income categories are significant. The relative risk ratios (RRR) for the two risk variables pull in favor of being previously insured for a lower degree of risk aversion (Strong to Moderate and Strong to Weak for Model 2:RP4 and from High to Low for Model 2:RP2). However, the magnitude of such effects is somewhat weaker relative to the same effects presented for the never insured in Table 3. Also for the previously insured, the introduction of risk preferences typically does not change the relative risk ratios and the significance levels of the control variables for both models (Model 2:RP4 and Model 2:RP2), relative to the benchmark model. The only exception matters for one of the age groups (60+ yrs.) in Model 2:RP2 (becomes significant at 5% level) and for one of the education groups (secondary education+) in both models (becomes insignificant). 

Finally, we conducted bivariate multinomial logistic regressions by regressing RP2 on enrollment status (see Appendix D). The relative risk ratios both for the never insured (RRR=2.21, *p=*0.11) and the previously insured (RRR=1.67, *p=*0.002) became somewhat weaker relative to the relative risk ratios presented in Table 3 (RRR=3.03, *p=* 0,098) and Table 4 (RRR=1.89, *p=*0.00).

## Discussion

Based on the BJKS instrument, our respondents on average envisage a relatively high degree of risk aversion. This finding is consistent with [3], surveying 300 smallholder farmers in Vietnam for eight different elicitation methods. Similar conclusions are reached by [7] who surveyed farmers in southern Vietnam. However, other studies reach different conclusions. A recent study by [21], taking place in the same region as the study by [7] performs a broad set of experimental measures of risk preferences. [21] found that the farmers were on average risk neutral and more risk tolerant than typical Western sample populations. The Vietnamese farmers were significantly less risk averse than American students and slightly more risk averse than Vietnamese students. Our sample is on average more risk averse than adult Norwegians and less risk averse than adult Chileans, while it does not differ much from the adult population of the USA. This last finding is somewhat surprising given that 75% of our sample is farmers – an occupation exposed to livestock and crop risks. On the other hand, in the USA, lower income people are living with a large background risk and the social network they can rely on for support may not be as good as in some developing countries.

Our results must also be evaluated in view of the elicitation methodology applied. We know that risk preference measurements vary across elicitation methodologies. [8] used a wide range of elicitation methods (eight) and found when examining consistency across methods, that the various measures were significantly correlated but weak. Furthermore, our lottery is a hypothetical one which implies that our results are stated rather than revealed. This means that, if using actual payments (payoffs), our measurement of risk preferences could have changed. [4] shows that the difference between an individual’s response to questions with and without payoffs increases with the size of payoffs.

Furthermore, the elicitation method might be unable to reflect the true risk preferences for other reasons as well. The majority of our respondents are farmers typically exposed to income risk (crop risk) and some of our respondents have low education or are without any formal education. Such factors imply that the respondents might be unfamiliar with the type of question raised by the BJKS instrument. On the other hand, we know that the BJKS instrument correlates well with different kinds of risk behaviors and hypothetical lotteries are necessary when considering large risks [37], as will be the case when considering health-related risks (quality of life, income and treatment expenditures).

Our analysis shows that the degree of risk aversion increases, to some extent, with a higher income for all three enrollment groups. Furthermore, higher risk aversion, measured by RP4, is significantly associated with higher income. This conclusion appears to be in line with other studies, for example [24–27] arrive identify weak positive (or absent) correlations between risk aversion and income. In contrast, [21] found strong negative correlations between risk aversion and income amongst Vietnamese farmers but no correlations with wealth**.** From theory, under certain assumptions, absolute risk aversion is decreasing and convex in wealth (see e.g. [43]).

There is also literature that discusses to what degree risk attitudes capture more than intrinsic preferences such as experiences, economic circumstances, and the environment. [33] is concerned with the ability and capacity to deal with shocks when markets are incomplete and uses wealth as a proxy for a household’s ability to deal with risks since wealthier households have better access to credit markets. In addition to credit markets, income shocks can be traded across time via transfers from family and friends, from having access to social networks and from adjusting the stock of assets. According to [33], the ability and capacity to deal with risks might induce lower risk aversion. [43] are concerned with sources of uncertainty that characterize the environment in terms of background risk. They find that higher background risks (income risk and liquidity constrained) induce a higher degree of absolute risk aversion. [37] the study, to what extent, welfare state generosity (protection against unemployment, sickness and medical expenditures) will reduce background risks and find that more extensive welfare states induce a higher average risk tolerance.

The above literature suggests that survey questions on risk preferences measures might capture individual preferences (tastes) as well as the ability and capacity to deal with risks and that risk preferences are endogenous in the sense that lower background risk (e.g. higher income and the existence of insurance markets) leads to lower risk aversion. For Tanzania, crop insurance might represent a device for coping with risk, however, such insurance is not very common and is most relevant for maize producers that typically are not located in the study area of our survey [44–47]. However, there are other mechanisms that potentially might impact the risk preferences of our sample. Examples are savings, the building-up of various assets (jewellery, land and livestock) and informal risk-management institutions that utilize social networks and kinship. Furthermore, choosing to be insured is a risk-coping strategy and such a choice might also impact risk preferences. If this is the case, a potential problem of reverse causation is introduced in our study. However, given such a mechanism, our odds-ratio estimates would be underestimated. [33], in her study on poor households in Uganda, simultaneously consider the effects of risk preferences and risk perceptions on agricultural production decisions. Our study on health insurance decisions has similarities since including three health-related variables (chronic disease, fear of future disease and EQ-5D health state). The three variables are self-reported and might represent subjective risk perceptions. Two of the three health related variables are insignificant while the chronic disease variable is significant in our analyses. However, omitting these variables introduces only minor changes in the relative risk ratios for the risk preference variables.

Our analysis confirms that the degree of risk aversion is higher for the insured relative to the uninsured and somewhat higher for the previously insured relative to the never insured. Furthermore, risk preferences are associated with the enrollment decision in the sense that moving from Strong to the next two categories (Moderate and Weak) has a significant effect while moving from Strong to Medium has insignificant effects (Model 1 and Model 2). These findings suggest that the difference in risk preferences, measured by BJKS, must be sufficiently high to be associated with the enrollment decision.

The literature on insurance and enrollment in LMICs is extensive and includes different designs and settings. Two systematic reviews [29, 30] are undertaken that include studies primarily from sub-Saharan Africa and Asia and where the outcome variable is binary (insured or uninsured). None of the reviewed studies (18 in [29] and 42 in [30]) include risk-preferences. [29] finds that higher income and positive perceptions towards health care quality and scheme leaders promote enrollment while [30] finds that enrollment increases with variables such as higher income, higher education and higher age. These conclusions correspond fairly well to our findings concerning the perception factors (quality and thrust) and age while the roles of income and education are partly different. For the logistic regression (Table 2), education is insignificant while higher income is significant only when moving from the poorest to the richest quintile. For the multi-nominal regressions (Tables 3 and 4), however, higher education promotes insurance, both for never insured and previously insured while higher income only matters for the previously insured. 

Our analyses identify insured and high-income earners as on average being more risk averse than the uninsured and low- and middle-income earners. However, the share of respondents belonging to the highest risk-averse category (Strong) is high in all income groups and all enrollment groups. For example, among the poorest, 63.1% are strongly risk averse while among the never insured, 63.3% are strongly risk averse. Hence, we are in a situation with seemingly strong risk preferences combined with a low enrollment rate (about 30%). There are several possible explanations for such a finding. First, the BJKS instrument might be unable to differentiate between respondents belonging to the highest risk aversion group, meaning that only a share of the respondents in this group possesses preferences significant enough to trigger enrollment. Second, the voluntary insurance scheme in question yields only partial coverage since providing protection primarily against outpatient treatment costs while some of the expensive services (inpatient services and medication) are not part of the benefit package unless being qualified for exemptions (elderly and children). Hence, the insured households are still confronted with significant risks. Third, despite a low enrollment rate (30%), the previously insured represent almost 54% of the total sample and together with the currently insured they amount to about 85% of the sample. Given this, one possible explanation might be that significant risk-averse preference promotes enrollment but other factors, such as adverse scheme experiences, induce households to withdraw from the scheme over time. 

Our analysis is clearly of importance since shedding light on the significance of risk preferences in connection with enrollment decisions in LMICs, however, this knowledge is difficult to transform into actual policies since risk preferences do not appear as a policy variable. However, in view of the significant risk preferences identified, a reduction in treatment-cost risks (an extension of the benefit package) might increase the net benefit from insurance, in this way promoting enrollment. It is not straightforward to compare the magnitude of the various estimated coefficients (odds ratios and relative risk ratios) in our analysis since the independent variables are measured differently and since some variables are categorical while others are not. However, besides risk-preferences, the perception variables appear as being important suggesting that policies that address quality of care, benefit-premium ratios, scheme leader trust and knowledge (traditional healers) might promote enrollment. In addition, income is relevant for being previously insured, relative to being insured, while income is not relevant for being never insured, relative to being insured. Our analysis might be relevant also in other aspects since our results remain surprisingly stable in response to the introduction of risk preferences. This finding suggests that the inclusion of risk preferences does not impact the relationships between the decision to enroll and other independent variables (control variables). As a consequence, former cross-sectional studies using household surveys from LMICs, that do not include risk preferences, might remain relevant.

## Conclusion

To the best of our knowledge, this paper is the first to study possible associations between risk aversion and enrollment into voluntary health-insurance schemes in LMICs using the BJKS elicitation method. It is also the first household survey in Tanzania that used the BJKS instrument to elicit people’s preferences. We identify strong associations between enrollment status and the degree of stated risk aversion among rural households in a region of Tanzania, in the sense that higher risk aversion increases the odds of being insured and reduces the odds of being uninsured (never insured or previously insured). A possible explanation for our findings is that individuals sort themselves in such a way that the more risk averse are enrolled into the scheme. A less likely explanation is that being insured increases the degree of risk aversion. Based on the literature on background risks, one would rather expect that lower health risks (treatment expenditures) would reduce the degree of risk aversion. Interesting topics for future research would be to assess the impact of being insured (lower health risks) on risk preferences and consider to what extent such changes impact other decisions that involve risks (spill-over effects) for example in terms of risky production choices (the adoption of new technologies).

Our findings confirm the presence of relatively strong risk-averse preferences when using the BJKS instrument to elicit such preferences. This finding is not necessarily surprising given the background risks typically present in developing economies. It is maybe more surprising that our sample, where the majority are smallholders from rural areas, is comparable to the sample from the USA when it comes to risk preferences. This raises the question as to whether the methods used to elicit risk preferences are valid for populations both in developed and developing economies and to what extent stated preferences are comparable across cultures and countries.

## Data Availability

The datasets used and/or analyzed during the current study are available from the corresponding author on reasonable request.

## Appendix A: Risk preferences and enrollment status

Table 8

**Table 8:** Risk preferences (RP4) by enrollment status (shares): Bivariate analysis

	Insured	Uninsured		Total	*p*-value
		Never insured	Previously insured		
***Risk preferences (RP4*****)**					
Weak	11.5	18.4	14.1	14.1	0.112
Moderate	3.2	9.2	7.9	6.7	
Medium	11.0	9.2	11.1	10.8	
Strong	74.3	63.3	66.6	68.4	

## Appendix B: Risk preferences and household income

Table 9

**Table 9:** Risk preference groups (RP4 and RP2) by household income (shares). Bivariate analysis

	Income3					
	Lower	Middle	High			*p*-value
*Risk preferences (RP4)*						
Weak	14.8	13.4	17.5	14.1		
Moderate	9.2	5.6	0.0	6.7		0.04
Medium	12.9	10.2	2.5	10.8		
Strong	63.1	70.8	80.0	68.4		
*Risk preferences (RP2)*						
Low	24.0	19.0	17.5	20.8		0.25
High	76.0	81.0	82.5	79.2		

## Appendix C: Risk preferences and household expenditures (socioeconomic status)

To construct the socioeconomic status (SES) variable, we used total household expenditures that were collected by asking respondents to state how they have spent on expenditures healthcare, food, and non-food items in the previous four weeks. The total household expenses were then divided into quintiles (20%) ranked from poorest to wealthiest (socioeconomic status). Expenditures are by some scholars preferred over income because people in the informal sector often have multiple income sources (a risk of measurement error). Furthermore, survey questions on household expenditures are less sensitive than questions on household income [48–50]. Table C1 shows the distribution of risk aversion across socioeconomic status. We observe that there are not any significant differences across risk categories across socioeconomic status. For RP4, however, there is a weak tendency for the two wealthiest socioeconomic groups to be somewhat more risk averse than the three least wealthy socioeconomic groups. The same tendency is prevalent when considering the dichotomous risk preference variable (RP2)

Table 10

**Table 10:** Percentage distribution of risk preferences (RP4 and RP2) by socioeconomic status (total household expenditures)

	Socioeconomic status (wealth quintiles)						
	Poorest	Poor	Average	Wealthy	Wealthiest	Total	*p*-value
Risk Preferences (RP4)							
*Weak *	15.2	13.2	15.2	13.2	13.9	14.1	
*Moderate*	9.7	6.3	7.6	4.9	4.9	6.7	
*Medium*	11.0	12.5	11.0	11.8	7.6	10.8	0.87
*Strong*	64.1	68.1	66.2	70.1	73.6	68.4	
Risk preferences (RP2)							
*Low*	24.8	19.4	22.8	18.1	18.8	20.8	0.57
*High*	75.2	80.6	77.2	81.3	81.3	79.2	

## Appendix D: Bivariate regressions

Table 11

**Table 11:** Bivariate logistic regression for risk preferences (RP2) on enrollment status (insured vs. uninsured)

Enrollment status	OR(95%CI)	P>z
Risk preferences (RP2)		
*Low*	1	
*High*	1.78 (1.16-2.73)	0.008***

note: ***, ** and * denote significance level (*p*-value) at 1%, 5% and 10% respectively

Table 12

**Table 12:** Bivariate multinomial logistic regression for risk preferences (RP2) on enrollment status (currently insured, previously insured and never insured)

Enrollment status	RRR (95% CI)	P>z
Base outcome = currently insured	0	
Never insured		
Risk preferences (RP2)		
*High*	1	
*Low*	2.21 (0.83-5.89)	0.114
Previously insured		
Risk preferences (RP2)		
*High*	1	
*Low*	1.67 (1.20-2.31)	0.002***

Note: ***, ** and * denote significance level (*p*-value) at 1%, 5% and 10% respectively

## Abbreviations

LMICs: Lower-middle income countries
UHC: Universal health coverage
BJKS instrument: Barsky, juster, kimball, and shapiro
CHF: Community health fund
iCHF: Improved community health fund
CBHIs-Community: Based health insurance schemes
MPL: The multiple price list
WHO: World Health Organization
RP (RP1, RP2, RP3, RP4): Risk preference variables
H-5: Reported- health state
ODK: Open Data Kit
USA: United states of America
OR: Odds ratio
RRR: Relative risk ratio
